# Early Maximal Strength Training Improves Leg Strength and Postural Stability in Elderly Following Hip Fracture Surgery

**DOI:** 10.1177/21514593211015103

**Published:** 2021-04-30

**Authors:** Ole Kristian Berg, Jens-Meinhard Stutzer, Jan Hoff, Eivind Wang

**Affiliations:** 1 Faculty of Health and Social Sciences, 5562 Molde University College, Molde, Norway; 2 Department of Orthopedic Surgery, 60494 Møre and Romsdal Hospital Trust, Molde Hospital, Molde, Norway; 3 Department of Circulation and Medical Imaging, Faculty of Medicine and Health Sciences, Norwegian University of Science and Technology, Trondheim, Norway; 4 Department of Physical Medicine and Rehabilitation, St. Olavs Hospital, Trondheim University Hospital, Norway; 5 The Exercise Clinic at Myworkout, Trondheim, Norway

**Keywords:** hip fracture, abduction, leg press, strength, balance

## Abstract

**Introduction:**

Hip fractures predominantly occur in the geriatric population and results in increased physical inactivity and reduced independency, largely influenced by a downward spiral of ambulatory capacity, related to loss of skeletal muscle strength and postural stability. Thus, effective postoperative treatment, targeting improvements in muscle strength, is sought after.

**Materials & Methods:**

Twenty-one hip fracture patients (>65 yr) were randomized to 8 weeks of either conventional physiotherapy control group (CG), or leg press and hip abduction maximal strength training (MST) 3 times per week. MST was performed applying heavy loads (85-90% of 1 repetition maximum; 1RM) and 4-5 repetitions in 4 sets. Maximal strength (bi- and unilateral 1RM), postural stability (unipedal stance test; UPS), and DEXA-scan bone mineral content/ density (BMC/BMD) were measured before and after the 8-week rehabilitation.

**Results:**

Both MST and conventional physiotherapy improved bilateral leg press 1RM by 41 ± 27 kg and 29 ± 17 kg, respectively (both p < 0.01), while unilateral leg press 1RM only increased after MST (within group and between groups difference: both p < 0.05). MST also resulted in an increase in abduction 1RM in both the fractured (5 kg, 95%CI: 2-7; p < 0.01) and healthy limb (6 kg, 95%CI: 3-9; p < 0.01), while no such improvement was apparent in the CG (between groups difference: p < 0.01). Finally, MST improved UPS of the fractured limb (p < 0.05). No differences were observed in BMC or BMD following the 8 weeks.

**Discussion:**

Early postoperative MST improved lower extremities maximal muscle strength more than conventional physiotherapy and was accompanied by improvements in postural stability.

**Conclusion:**

Implementing MST in early rehabilitation after hip fracture surgery should be considered as a relevant treatment to curtail the downward spiral of reduced ambulatory capacity typical for this patient group, possibly reducing the risk of recuring falls and excess mortality.

**Trial Registration:**

https://clinicaltrials.gov/ct2/show/NCT03030092

## Introduction

Hip fractures are a common and serious health challenge, predominantly affecting the geriatric population, with a skewed distribution between sexes in disfavor of females.^1^ In addition to the acute pain and debilitation, people suffering a hip fracture have a substantial long-term loss of functional status.^2^ The attenuated physical function following a hip fracture also result in a severe challenge for elderly to remain independent. Indeed, as much as 43% of hip fracture patients lost the ambulatory capacity to move outside their own home unassisted, and as much as 56% lost their ability to walk unaided.^3^ Detriment of skeletal muscle strength during the post fracture period has been found to indicate a worse mobility recovery, underscoring the importance of improving lower limb muscle strength postoperatively.^4^

Maximal strength training (MST), which consists of few repetitions (4-5) at heavy loads, ∼85-90% of 1 repetition maximum (1RM), with a focus on maximal mobilization of force in the concentric phase, has previously proven to be feasible and effective for strength and functional gains early in the postoperative phase following elective total hip arthroplasty.^5,6^ Thus, a similar intervention may be similarly favorable following hip fracture surgery. The age and frailty were somewhat lower in these previous investigations, compared to what is typical for hip fracture patients. However, in a paper by Overgaard, Kristensen^7^ progressive strength training with a somewhat lower strength training intensity than MST (15-10RM) in the early postoperative phase, after hip fracture, was documented to be feasible. Importantly, in the Overgaard, Kristensen^7^ study, the strength training led to substantial improvements in ambulatory capacity, postural stability and reduced hip fracture-related pain. MST is documented to be more effective in improving muscle strength and force generating capacity than conventional strength training,^8,9^ such as in the study by Overgaard, Kristensen.^7^ Therefore, MST may be a relevant approach also in the postoperative phase for hip fracture patients, given that it is feasible to use such heavy loads shortly after hip fracture surgery.

To address the question of the relevance and feasibility of MST in physical rehabilitation of hip fracture patients in the early postoperative phase, we conducted a study comparing physical rehabilitation treatment as usual to leg press and abduction MST initiated ∼1 week after discharge from hospital. We hypothesized that 1) MST would increase muscle strength and postural stability more than treatment as usual, and that 2) MST would be feasible in the patient group.

## Methods

### Study Design

This study was designed as a randomized controlled trial. After obtaining written informed consent in the hospital ward post-surgery, subjects were randomly allocated to an intervention group (MST) or a control group (CG), and followed for 8 weeks. The pre-test was conducted during the first week after discharge from the hospital, after which, subjects were followed for 8 weeks of conventional physiotherapy for the CG or MST in the intervention group. Inclusion criteria were age > 65 yrs. and traumatic hip fracture that was treated surgically. Exclusion criteria were cognitive impairment affecting ability to grant informed consent, pre-existing handicap that impacted ability to perform tests and training, COPD stage > 2, heart failure NYHA stage > 2, multiple sclerosis and myopathy. All hip fracture classifications and surgical treatments were eligible for inclusion. The study was approved by the Norwegian Regional Committee for Medical and Health Research Ethics, and all parts of the study were performed according to the Declaration of Helsinki. Clinical trials registration NCT03030092.

### Testing Procedures

Before and after 8 weeks of conventional physiotherapy or experimental treatment with MST, subjects reported to the radiology laboratory and rehabilitation clinic on separate days for pre-testing. On day 1, a Dual-Energy X-ray Absorptiometry (DEXA) scan was conducted to assess bone mineral content (BMC) and density (BMD), height and weight was recorded. On day 2 (16 ± 6 days postoperatively), postural stability and muscle strength was assessed. Fracture classification, weight bearing status (WB-status) and surgical treatment were recorded from the hospital medical records. The WB-status postoperatively for the injured limb was defined as either full loading, loading until pain threshold, or touch loading. Integrity of the operated hip was assessed by x-ray and physical examination after the 8-week intervention, as part of a 3 months in-hospital follow-up appointment.

#### Dual-energy X-ray absorptiometry

Subjects reported to the radiology laboratory for DEXA-scans at the same time of day prior to and after the 8-week physical rehabilitation period. The BMC and BMD at the lumbar spine (L1-L4) was assessed using a Medix DR (Medilink, Montpellier, France) with the subject laying in the supine position, with the hips and knees flexed, supported by a positioning cushion (height 30 cm), to flatten the lordosis of the lumbar spine. Healthy limb femoral neck BMC and BMD was assessed in the prone position with feet fixated on a foot rest.

#### Postural stability

Prior to strength testing, postural stability was assessed by the unipedal stance test (UPS) with eyes open, as previously described by Springer, Marin, Cyhan, Roberts, Gill.^10^ Briefly, subjects were positioned within a parallel bars stationary walking support with a physical therapist behind them and the examiner recording the time kneeling in front of them. The dominant leg was determined as the leg the subject would have chosen to kick a ball with prior to the fracture. The test was carried out on both legs. No shoes were worn to standardize pre- and post-test. The longest recorded stance time, for each leg, from 3 trials was noted as the UPS.

#### Strength measurements

Both bilateral and unilateral leg press 1RM were assessed using a horizontal seated leg press (Isotonic line white, Technogym, Italy). Bilateral leg press was assessed first. Following a warm up of 2 light to moderate sets of 10 repetitions, the load was increased in increments of 10 kg until failure to perform the leg press as instructed. The backrest was at ∼45° to the horizontal plane. The initial concentric action was assisted to position the legs near full extension, subjects were then asked to conduct a controlled eccentric movement until the angle between the tibia and femur was 90°. From this position, an unaided concentric movement was performed to extend the legs back to the initial position. The highest load that could be lifted as instructed was recorded as 1RM. After a 5-minute break, the unilateral 1RM of the healthy limb was recorded using the same procedure, with the fractured limb resting on a platform attached to the seat in the horizontal plane.

Following a 10-minute break subjects were positioned in the parallel bar walking support and a sling was attached around the ankle and connected to the cable of a cable-pulley system. From a neutral standing position with feet ∼10 cm apart, subjects were instructed to abduct their leg from 0° to 25° in the hip joint while taking care to keep toes pointing forward and not moving the upper body. A physical therapist was positioned behind the subjects observing if upper body involvement was present and that only light finger touch on the parallel bars was used as a reference, to maintain balance. A stool was laid on the floor to serve as a target at 25° abduction. The examiner recording the test was positioned in front of the subject and was monitoring that the legs were straight (no knee flexion), toes were pointing forward, and that the target 25° abduction was reached. As with the leg press 2 sets of light to moderate warm up of 10 repetitions was conducted prior to gradual increase of load by 0.5 kg until failure to comply with test instructions. After recording the 1RM abduction strength in 1 leg, a short rest was given prior to conducting the same test with the other leg. The abduction strength and UPS results from pre- and post-test were pooled to assess the relationship between abduction strength and postural stability.

### Maximal Strength Training

The MST intervention group trained 3 times per week for 8 weeks at a physical rehabilitation center, supervised by a physical therapist with training in administering MST. Each visit consisted of the same warm up and MST exercises. Prior to the MST, as a warm up, the patients performed 5 minutes of light cycling on an ergometer (Cardio Care 927 E, Monark, Sweden) followed by 2 sets of high knee raises, sideways walking and heel raises in the parallel bars, and finally 2 x 10 chair rises. Subsequently, bilateral leg press MST was conducted. Subjects started training at ∼85% of pre-test 1RM and conducted 4-5 repetitions in the same range of movement as for the 1RM test in 4 sets. When 5 repetitions could be completed in all 4 sets, the weight was increased by 5 to 10 kg in the next session. To maximally stimulate efferent neural drive, the subjects were encouraged to conduct the concentric phase as fast and forcefully as possible. Following the leg press MST, leg abduction MST was conducted under the same conditions as for the 1RM test, with the exception of only 1 physical therapist being present. Subjects alternated between the right and left leg between each set, for a total of 8 sets (4 on each leg). As with the leg press, subjects started training on ∼85% of leg specific pre-test 1RM, and the weight was gradually increased by 0.5 to 1 kg after each session where 5 repetitions could be completed in all of the 4 sets for a given leg. Each MST session lasted approximately 30 minutes.

### Conventional Physiotherapy

Subjects in the control group received treatment as usual for outpatients following hip fracture, for a duration of 8 weeks. Mainly consisting of unloaded and body weigh loaded exercises targeting range of motion and activities of daily living, e.g. prone hip flexion of the fractured limb, unloaded hip extension and abduction, chair rises and stair climbing. The conventional physiotherapy was offered both in rehabilitation clinic and as home rehabilitation 2-3 times per week. Each CG session lasted approximately 40 minutes.

### Statistical Analysis

To assess the difference between physical rehabilitation using MST or treatment as usual, analysis of covariance was applied, with outcome after treatment as the dependent variable, and baseline value and treatment group as covariates.^11^ IBM SPSS statistical software (version 25) was used for statistical analysis. Variables were assessed for normality distribution by the Shapiro-Wilk test, within group change was analyzed using paired sample T-test for normally distributed variables, or Wilcoxon signed ranks test for variables that were not normally distributed. Pearson’s correlation was used to analyze the correlation between UPS and abduction strength. Feasibility was determined as ability to perform 90% of MST sessions and no adverse events related to the operated hip. For all analyzes, the level of significance was set to p < 0.05. Data are presented in text and tables as means ± SD, or as mean difference with 95% CI in the case of between group differences, and means ± SE in figures for clarity.

## Results

### Subject Characteristics

**Figure 1.**
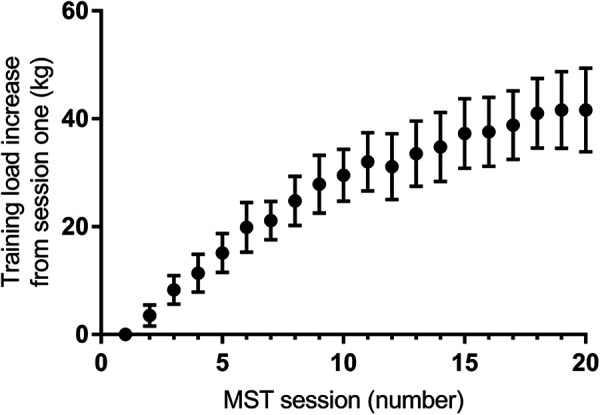
Average progressive training load increment per MST session in bilateral leg press from the training load lifted during the first MST session. Graph illustrates sessions 1-20 in the leg press exercise, which were completed by all subjects in the MST group (n = 8). Values are mean ± SE.

Two patients dropped out during pre-testing, reporting inconvenience and lack of motivation as reasons. Three dropped out of the MST group during the intervention, due to; discomfort from preexisting inguinal hernia during training, readmission to hospital for somatic disease unrelated to fracture or training, and undernourishment likely due insufficient nutritional intake. Baseline characteristics of the 18 subjects that completed the study are presented in Table 1. Subjects in the MST-group completed 23 ± 2 of the planned 24 sessions (96% compliance). The subjects appeared to tolerate MST without any adverse effects on the operated hip, and training load increased gradually as expected (Figure 1).

**Table 1.** Subject Characteristics.

	MST	CG
Age, yr	74 ± 9	75 ± 11
Height, cm	171 ± 5	166 ± 8
Weight, kg	67 ± 10	68 ± 10
Sex, female/male	4/4	8/2
Grip strength, right/left, kg	25 ± 9/ 24 ± 9	24 ± 6/ 22 ± 7
Fractured leg, dominant/ non-dominant	4/4	5/5
WB-status, full/ pain threshold/ touch	4/2/2	7/2/1

Values are mean ± SD. Maximal strength training group (MST) n = 8, control group (CG) n = 10.

### Leg Strength and Postural Stability

**Figure 2.**
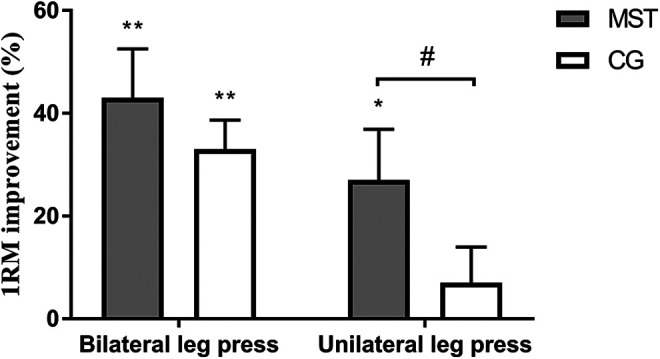
Percent improvement of leg press strength following 8 weeks of maximal strength training (MST) or conventional physiotherapy in control group (CG). Values are mean ± SE. Within group change from pre-test * p < 0.05, **p < 0.01. Between group effect # p < 0.05.

Both the MST and CG increased bilateral leg press 1RM by 43 ± 27 and 33 ± 18% respectively during the 8 week intervention (p < 0.01 for both groups), no between group effect was observed. On average, the pretest bilateral leg press was 139 ± 42% of the subjects’ bodyweight. Unilateral leg press 1RM increased by 13 kg (CI: 1 to 24) more in the MST group compared to the CG, and only in the MST group a significant improvement (p < 0.05) was observed (Figure 2 and Table 2). The fraction of the bilateral 1RM explained by the healthy unilateral 1RM decreased from 56 ± 13 to 49 ± 11% and 67 ± 22 to 52 ± 10% for MST and CG respectively (both p < 0.05). Abduction 1RM increased by 5 kg (CI: 2 to 7) and 6 kg (CI: 3 to 9) more in the MST group compared to CG (p < 0.01) for the fractured and healthy limb respectively. For both legs, only the MST group significantly (p < 0.01) increased their abduction strength from pre to post-test (Table 2). The MST group also improved the postural stability assessed by UPS on the fractured limb following the intervention (p < 0.05). The CG did not significantly improve their postural stability, and no between group effect was observed. There was a significant correlation between abduction 1RM and UPS in both the healthy (r = 0.449 p < 0.01) and fractured leg (r = 0.515 p < 0.01).

**Table 2.** Maximal lower extremity strength, postural stability and spine bone mineral content/density.

	MST			CG		
	PRE	POST		PRE	POST	
Bilateral leg press 1RM, kg	100 ± 43	141 ± 62	**	92 ± 35	121 ± 43	**
Unilateral leg press 1RM, kg	56 ± 29	71 ± 39	* #	56 ± 19	58 ± 18	
Abduction 1RM—fractured limb, kg	6.9 ± 4.0	12.4 ± 5.1	** ##	4.4 ± 3.9	5.5 ± 3.1	
Abduction 1RM—healthy limb, kg	7.4 ± 3.1	13.4 ± 5.0	** ##	8.5 ± 4.2	8.2 ± 3.6	
UPS—fractured limb, s	8 ± 16	17 ± 18	*	2 ± 3	12 ± 18	
UPS—healthy limb, s	14 ± 18	22 ± 20		17 ± 17	21 ± 18	
Spine BMC, g	61.31 ± 18.61	61.80 ± 18.66		58.74 ± 21.25	58.37 ± 21.72	
Spine BMD, g/cm^2^	0.907 ± 0.186	0.913 ± 0.187		0.952 ± 0.221	0.953 ± 0.227	

Abbreviations: 1RM, 1 repetition maximum; UPS, uni pedal stance-test. MST (n = 8), CG (n = 10). Values are mean ± SD. Within group change from pre-test * p < 0.05, **p < 0.01. Between group effect # p < 0.05, ## p < 0.01.

### Fracture and Bone Health

The fractures by group (MST/CG) were: medial collum 6/7, pertrochanteric 0/2, intertrochanteric 0/1, subtrochanteric 1/0, and lateral collum 1/0. Surgical treatment by group were: total prosthesis 2/1, hemi prosthesis 1/4, intermedullary nailing 2/3, and screws 3/2. WB-status is reported in Table 1. No indications of compromised hip integrity was apparent from the post-intervention x-ray and physical exam in either MST or CG. Four patients (MST/CG: 2/2) had previous hip surgery on the contralateral hip, thus DEXA results for femoral neck were not analyzed. No significant change in BMC or BMD of the spine (L1-L4) was observed for either of the groups following the intervention (Table 2).

## Discussion

Hip fractures are associated with substantial acute strength deficit in the lower extremities,^7,12^ leading to reduced ambulatory capacity,^3^ and frailty in elderly. Physical training interventions that efficiently abate the loss of function related to muscle weakness are sought after. The current study investigated the effect of leg press and abduction MST, tailored to maximize strength gains in the early postoperative phase after hip fracture. The main findings were that 8 weeks of postoperative MST led to a larger increase in leg abduction and unilateral leg press strength, accompanied by a larger improvement in postural stability, compared to conventional physiotherapy. Moreover, despite applying heavy loads (85-90% of 1RM), MST was feasible for the patient group. MST appears to be a superior alternative to conventional physiotherapy, for improvement of lower extremity strength and postural stability, in the early postoperative phase for elderly hip fracture patients.

### Lower Extremity Maximal Strength and Ambulatory Capacity

Lower extremity maximal strength increased more after MST compared to conventional physiotherapy, evident as a larger increase in unilateral leg press 1RM and abduction 1RM. Notably, the 43% increase in bilateral 1RM following early postoperative MST in the current study was, despite the frailty of the elderly hip fracture patients, a similar percentage improvement to what has been previously reported for 8 week MST interventions with 3 sessions per week in both young (50%)^8^ and healthy elderly (68% and 53%).^9,13^ Moreover, the MST improved unilateral leg press 1RM by 27%, resulting in a maximal strength higher than baseline level. No improvement of unilateral leg press 1RM was observed in the CG. This may have important clinical relevance when the contralateral limb strength is impaired, following e.g. hip fracture surgery, because unilateral strength gains are accompanied by improved neuromuscular function in the contralateral limb.^14^ These strength gains are likely further of clinical relevance, as quadriceps strength has been found to be a robust predictor of post hip fracture walking and stair climbing speed, as well as the prevalence of falls.^15,16^ Muscle strength in the lower extremities is also a limiting factor influencing the ability to rise from a chair, which is an important task to master for independency in frail elderly.^17^

A common challenge for gait function following hip surgery is limping. The strength and cross sectional area of hip abductor muscle has been associated with postoperative limping following hip surgery.^18,19^ Moreover, hip abductor strength appears to be a key predictor of hip fracture patients’ ambulatory capacity.^20^ Thus, the superior effect of MST on hip abduction 1RM reported herein further accentuate the relevance of MST in postoperative physical rehabilitation after hip fracture surgery. Results from a period of MST following total hip arthroplasty surgery indicate that the superior effect of MST persist at both 3 and 6 months postoperatively.^6^ This rapid and potent effect on muscle strength from MST in hip fracture patients is likely of great importance to curtail a potential life threatening downward spiral of inactivity and impaired functional capacity following hip fracture.^2^ Indeed, it has even been indicated that the excess mortality following hip fracture in elderly women is not explained by pre-fracture medical conditions.^21^ Coupled with reports that cardiovascular disease is the main cause of early death in this patient group, removing limitations to physical activity is paramount in targeting the excess mortality.^22^

### Postural Stability, Implications for Falls and Fractures

Older individuals that have poor balance are more prone to falls and fractures, and the vast majority of hip fractures are caused by falls from standing height, and mostly occur in the patients’ residence.^23^ Indeed, in the current study, impaired balance was evident in the patient group, as healthy limb UPS score prior to rehabilitation (Table 2) was 27% lower compared to normative data typical for their age group (average score for 70-79 year old’s; ∼22 seconds).^10^

Additionally, following a hip fracture the risk of experiencing a second debilitating fall is increased.^24^ This is certainly evident from the severely impaired postural stability, UPS, measured in the fractured limb after hip fracture surgery (MST: 8 seconds, CG: 2 seconds. Table 2). Previous studies administering strength training in physical rehabilitation of hip fracture patients have reported improved balance measured with the Berg Balance Scale and the static tandem test.^7,25^ Following 8 weeks of postoperative training in the current study MST resulted in a prolonged UPS of 17 seconds in the fractured limb, implying a substantial risk reduction for recurring falls.

An important component of the improved postural stability following MST may be the hip abduction strength. Indeed, Wilson, Robertson, Burnham, Yonz, Ireland, Noehren^26^ reported that hip abduction strength correlated (r = 0.409) with performance in the Y Balance Test. Similarly, our data displayed a correlation between abduction 1RM and UPS in both the healthy limb (r = 0.449) and the fractured limb (r = 0.515). Taken together, these findings underscore the importance of targeting hip abduction strength during early postoperative exercise training, to improve postural stability and decrease the risk of recurring falls and injuries.

### Feasibility of Maximal Strength Training in Elderly Hip Fracture Patients

No adverse events related to the operated hip occurred following MST in the frail and newly operated elderly hip fracture patients. Importantly, each subject was assessed by an orthopedic surgeon postoperatively and cleared for inclusion. This included granting waiver from postoperative loading or hip-flexion restrictions during testing and training. Restrictions were maintained outside the laboratory and training facilities.

Progressive strength training with somewhat lower loads (10RM) than what is used for MST (4-5RM) has previously been shown to be feasible in elderly hip fracture patients.^7^ Kronborg, Bandholm, Palm, Kehlet, Kristensen^12^ even demonstrated that strength training was feasible in the hospital ward 2.4 days after hip fracture surgery. However, this was demonstrated for the knee-extension exercise, which does not load the hip joint. More comparable to the current investigation, Overgaard, Kristensen^7^ added the leg press exercise in addition to knee-extension, starting 17.5 ± 5.7 days postoperatively. Likewise, the 1RM test that initiated the training in the current study was conducted 16 ± 6 days postoperatively. Training loads as high as 80% of 1RM have previously been utilized in hip fracture patients.^25^ However, in that study the training started 84 days postoperatively.

The compliance to training sessions was high in the MST group (96%). This is comparable to what has been reported in healthy age matched subjects (92 and 86%) conducting the same volume of MST over 8 weeks.^9,13^ Interestingly, it is very similar to the 95% compliance reported to progressive strength training shortly after hip fracture surgery by Overgaard, Kristensen.^7^ Of note, the Norwegian health care system cover transportation to and from rehabilitation, and transport was scheduled for the patients when appropriate. This likely reduces barriers to complying with appointments. Nonetheless, the compliance was above 90% and with no adverse events related to the operated hip MST in the early postoperative phase following hip fracture surgery should be considered feasible.

It should be noted from the dropouts that leg press MST may be uncomfortable and cause patients to discontinue training if they have an inguinal hernia. The incidence of 1 subject becoming undernourished during the intervention underscores the intertwinement of health related factors that influence rehabilitation, especially in a geriatric population. Indeed, impaired nutritional status is common after hip fracture.^27^ Thus, therapists working with physical rehabilitation should be part of a multidisciplinary team communicating necessary measures to ensure adequate dietary intake.^27^

### Study Limitations

Comparing a specific intervention, such as MST, to treatment as usual needs to be considered in light of the heterogeneity in exercises, frequency and dosage that is utilized in conventional physiotherapy. As treatment may vary between different clinics and therapists, a relevant criticism to this design is the variability within the control group. Thus, grounds for concluding that MST is more effective than any given specific alternative is lacking. However, the design grants the basis to exemplify the potential effect of MST compared to the expected average effect of treatment that is currently offered. This may be relevant when seeking to evaluate if an intervention should be considered implemented in clinical practice.

The random group allocation in the current investigation yielded a somewhat skewed sex distribution between groups. Notably, there were no significant differences in subject characteristics between groups. Moreover, there were no baseline differences in the outcome variables between groups. However, sex differences in response to training may have occurred.

In relation to risk of falling and ambulatory capacity, dynamic balance tests and analysis of gait function may be more sensitive than the static balance test utilized, and should be implemented in future studies. However, static balance has been associated with increased risk of falling and functional performance in elderly.^28,29^

### Clinical Implications

The superior effect of MST compared to conventional physiotherapy with regard to maximal lower extremity strength and postural stability, should entice clinicians to recommend implementation of MST in rehabilitation shortly after hip fracture surgery. The leg press exercise should be considered well suited as it is performed in a stable semi recumbent position allowing focus to be put on the force generation by removing the challenge of impaired balance. Although, MST and heavy loading may raise concerns of compromising the integrity of the operated hip, the current study revealed no such adverse effects. Furthermore, it should be highlighted that leg press 1RM was in fact only slightly above bodyweight at pretest. Thus, although terms as maximal and heavy may not immediately resonate with frail elderly hip fracture patients, the relative load to individual strength should be emphasized.

Although, no subjective measures of function, fear avoidance or quality of life was recorded, anecdotal comments from subjects may be of relevance. Specifically, several subjects mentioned after the pretest 1RM leg press test that it made them feel safer about loading the hip during everyday tasks that they had abstained from doing in fear of pain or damage to the hip. Thus, apart from the physical benefits of MST demonstrated herein, it may hold psychological benefits and reduce barriers in daily life after surgery.

## Conclusion

This study revealed that 8 weeks of hip abduction and leg press MST performed shortly after surgery improved lower extremities muscle strength and postural stability more than conventional physiotherapy. The improved postural stability was likely impacted by the increased hip abduction strength. Moreover, a high compliance with the MST program was found, and no adverse events related to the operated hip was reported. Taken together, this indicates that MST is a feasible treatment in the early postoperative phase following hip fracture, and should be considered implemented in the overall postoperative rehabilitation plan, to reduce the risk of recurring falls, and improve ambulatory capacity.
